# Editorial: New Macrocycles and Their Supramolecular Perspectives

**DOI:** 10.3389/fchem.2020.00128

**Published:** 2020-02-26

**Authors:** Carmine Gaeta, De-Xian Wang

**Affiliations:** ^1^Laboratory of Supramolecular Chemistry, Department of Chemistry and Biology “A. Zambelli”, University of Salerno, Fisciano, Italy; ^2^Beijing National Laboratory for Molecular Sciences, CAS Key Laboratory of Molecular Recognition and Function, Institute of Chemistry, Chinese Academy of Sciences, Beijing, China; ^3^School of Chemical Sciences, University of Chinese Academy of Sciences, Beijing, China

**Keywords:** biomimetic systems, biomedical applications, supramolecular catalysis, cucurbiturils, cycloparaphenylenes, carbon-nanohoops, pillararene, chiral recognition

After more than half a century from the discovery of crown-ethers by Pedersen, macrocyclic hosts continue to be protagonists in supramolecular chemistry. Their peculiar structures make them ideal candidates to perform supramolecular functions such as catalysis, molecular and biomolecular recognition, sensing, self-assembly, and threading to give interpenetrated architectures. In addition, thanks to their synthetic versatility, the macrocycles are useful platforms for the design of more elaborated structures for the self-assembly of supramolecular polymers and for applications in biomimetic chemistry. These aspects have stimulated the creativeness of the scientists that in this way started imagining novel macrocyclic structures with the aim to perform ever more advanced supramolecular functions and properties. Thus, in the last years, in addition to the most innovative aspects regarding the “old” macrocycles, much attention has also been focused on the synthesis of new macrocycles. These studies have led to the discovery of novel classes of hosts such as pillararenes, cycloparaphenylene, biphenarenes, oxatubarene, large resorcinarenes, and a wide class of heteracalixarenes, coronarenes, which have found applications in several areas of supramolecular chemistry.

Starting by these considerations, we organized this article collection in which a considerable attention was devoted to the study of *new macrocycles*, and their applications in the field of molecular recognition and biological application, biomimetic chemistry, including supramolecular catalysis in nanoconfined spaces.

Regarding the biological applications, the review by Hadrovic et al. shows the potentialities of water-soluble molecular tweezers (MTs) which are able to complex the cationic side chains of lysine and arginine inside their cavities. The complexation is driven by secondary interactions between the cationic parts and the electron rich aromatic cavity of MTs. Interestingly, the inclusion of the cationic side chains of biologically-relevant amino acids inside the cavity of MTs prevents the pathologic protein aggregations. The toxic aggregates of protein species are dissolved and redirected to form amorphous, benign assemblies. Thus, MTs can be considered as promising candidates for disease-modifying therapy in early stages of neurodegenerative diseases.

In the last decades many efforts have been directed toward the design of macrocyclic derivatives with applications in the biomedical field. Das et al. show that the cucurbit[n]urils (CBs) are ideal candidates for applications in the area of medicinal-chemistry and chemical-biology thanks to their low toxicity and host-guest properties. In particular, CBs are able to encapsulate drugs for their formulation and delivery and finally show interesting properties in the controlled drug-release. The host-guest complexes of CBs have found interesting applications for sensing, diagnostic, theranostic, and other relevant medicinal or bioanalytical applications.

The exploration of ever-new biomedical applications led to metal-calix[4]arene complexes able to inhibit the growth of bacteria, fungi, and cancerous tumor cells. As described by Noruzi et al., the biological activity is ascribable to the inorganic ions rather than calixarene ligand.

In the field of biomedical applications of macrocyclic compounds, an interesting review has been reported by Mostovaya et al. The authors show some examples in which the PLA has been modified with various macrocyclic fragments to obtain derivatives with promising properties for drug-delivery systems, photosensitizers in photodynamic therapy, protein binding and biosensing.

Anions play important roles in a wide range of natural and biological processes. Thus, the development of synthetic molecules designed to mimic the efficiency of the natural anion-receptors is an intensively active area of research in supramolecular chemistry. In their work, Miranda et al. report the synthesis of two bidentate dihomooxacalix[4]arene receptors bearing phenylurea moieties substituted with electron-withdrawing groups at the lower rim via a butyl spacer. The binding affinity of these receptors toward several relevant anions was investigated and some of them were also studied as ditopic receptors for organic ion pairs, namely monoamine neurotransmitters and trace amine hydrochlorides.

Supramolecular chemistry is always inspired by biological systems and therefore biomimicry plays a crucial role in the design of novel macrocyclic hosts. Among the natural supramolecular systems, Gramicidin A (gA) is a natural peptide channel with a well-established, simple structure, and function: cations and water are transported together along the channel. In their review, Sun and Barboiu report examples of synthetic compounds able to mimic the functions of the natural gA. These systems are channel-type superstructures formed by self-assembly and provide remarkable combinations of functions similar to gA channel: water permeability, proton conductance via Grottus mechanism, cation vs. anion selectivity, single-channel activity.

In the context of the biomimetic systems, the self-assembled hexameric resorcinarene capsule shows peculiar features that make it an efficient biomimetic catalyst for organic reaction. Like a natural enzyme, the resorcinarene capsule shows an internal cavity able to host complementary guests, and able to accelerate the reactions by (i) nano-confinement effect; (ii) stabilization of intermediates and/or transition states. Gambaro et al., in their research article show that the hexameric resorcinarene capsule is able to catalyze the formation of bis(heteroaryl)methanes by reaction between pyrroles or indoles and carbonyl compounds (α-ketoesters or aldehydes) in excellent yield and selectivity. The authors suggested that the capsule can play a double catalytic role as a H-bond catalyst, for the initial activation of the carbonyl substrate, and as a Brønsted acid catalyst, for the dehydration of the intermediate alcohol.

In natural systems, the receptor-substrate association takes account of the chirality of the individual components and consequently the preference of a receptor for the given substrate and vice versa, depends on the spatial relationships between the individual interacting species. Taking inspiration from this, many scientists focused their attention on the study of host-guest interactions between chiral species. Guo et al. synthesized a couple of water-soluble chiral 2,6-helic[6]arene macrocycles and showed that they form stable 1:1 complexes with fluorescent cationic pyridinium guests in water. Compared with the free guest, the host-guest complex exhibited enhanced fluorescence. The host-guest complexes between 2,6-helic[6]arene and the cationic pyridinium guest were self-assembled in water to obtain supramolecular aggregates which showed rectangular or hexagonal nanostructures by SEM images. Interestingly, it was found that the assemblies showed clear mirror-image CD and CPL spectra in aqueous solution, which revealed a consecutive chirality transfer from the chiral macrocycle to the achiral guest.

Regarding the chiral recognition, in their paper, Gangemi et al., show that a chiral fluorescent-uranyl salen host acts as a receptor for the enantiomeric recognition of α-aminoacids derivatives, with high association constants and an excellent enantiomeric discrimination between the two enantiomers of phenylalanine.

The design and the synthesis of chiral macrocyclic hosts has attracted much attention, and therefore the planar chirality shown by pillar[n]arenes was considered very useful for chiral molecular recognition, chirality switches, and catalysis. The planar chirality of pillar[n]arenes is mainly caused by the inherent substitution pattern of the aromatic units. Usually, the synthesized pillar[5]arenes are racemic mixtures and racemization takes place by rotation of its aromatic units. In their work, Sun et al. show that the introduction of β-galactose units on both the rims of the pillar[5]arene prevents the racemization according to dynamic ^1^H NMR studies. After separation, the two stable diastereoisomers (*Sp-D*)-**GP5** and (*Rp-D*)-**GP5** were able to capture a guest molecule (DNS-CPT) to form a host-guest supramolecular amphiphile. This can further self-assemble into chiral nanoparticles with the *Sp* and *Rp* planar chirality of (*Sp-D*)-**GP5** and (*Rp-D*)-**GP5** still being retained, suggesting GP5 could be as reliable chiral sources to transfer the Sp and Rp planar chirality.

The research for new macrocyclic structures often brings to the discovery of new recognition motifs. Thus, in the last years the cycloparaphenylene (CPP) macrocycles and π-extended carbon-nanohoops have attracted much attention due to their intriguing abilities to establish π···π and cation···π interactions with complementary guests. In their review, Lu et al. highlight the supramolecular properties of CPPs and π-extended carbon-nanohoops mainly focusing on the size-selective encapsulation of fullerenes, endohedral metallofullerenes, small molecules, and CPP-based mechanomolecules.

Regarding the synthesis of mechanomolecules, Zhang R. et al. reported the synthesis of pillar[5]arene-based [1]rotaxanes. Thus, a series of amide-linked pillar[5]arene-based [1]rotaxanes with ferrocene unit as the stopper were obtained. Interestingly, the synthesized monofunctionalized pillar[5]arenes show a self-inclusion property, which gives rise to a pseudo-rotaxane, being the length of the imine chain the key role in this process. A rotaxane is formed through amidation of a ferrocene dicarboxylic acid which acts as a plug. In addition, due to the ferrocene units, the pillar[5]arene-based [1]rotaxanes exhibit electrochemically reversible property.

The fascinating structure of pillararenes and their amazing supramolecular properties are stimulating the imagination of many scientists. In their work, Jia et al. report acyclic pillar[n]naphthalene (*n* = 2–4, dimer, trimer, and tetramer) oligomers, which are made up of 2,3-diethoxynaphthalene units linked by methylene bridges. The crystal structure of the tetramer shows an interesting pseudo-cycle-shaped structure in the solid state. The oligomers show interesting recognition properties toward cationic guests.

Among the new macrocycles, the heteracalixaromatics or heteroatom-bridged calix(het)arenes have gained a role of primary importance thanks to their unique conformational features and versatile recognition properties. In their work, Zhang E-X. et al. synthesized a number of hydroxyl-substituted azacalix[4]pyridines using Pd-catalyzed macrocyclic “2+2” and “3+1” coupling methods and a protection-deprotection strategy of hydroxyl group. The conformational properties of these hydroxyl-substituted azacalix[4]pyridines have been studied both in solution and in the solid state by X-ray analysis. Taking the hydroxyl substituted azacalix[4]pyridines as molecular platforms, multi macrocycle-containing architectures and functional building blocks were then constructed.

## Author Contributions

CG drafted the work. D-XW revised it critically.

### Conflict of Interest

The authors declare that the research was conducted in the absence of any commercial or financial relationships that could be construed as a potential conflict of interest.

